# Strong fascin expression promotes metastasis independent of its F-actin bundling activity

**DOI:** 10.18632/oncotarget.22249

**Published:** 2017-11-01

**Authors:** Lisa S. Heinz, Stefanie Muhs, Johanna Schiewek, Saskia Grüb, Marcus Nalaskowski, Yuan-Na Lin, Harriet Wikman, Leticia Oliveira-Ferrer, Tobias Lange, Jasmin Wellbrock, Anja Konietzny, Marina Mikhaylova, Sabine Windhorst

**Affiliations:** ^1^ Department of Biochemistry and Signal Transduction, University Medical Center Hamburg-Eppendorf, Hamburg, Germany; ^2^ Institute of Tumor Biology, University Medical Center Hamburg-Eppendorf, Hamburg, Germany; ^3^ Department of Gynecology, University Medical Center Hamburg-Eppendorf, Hamburg, Germany; ^4^ Institute of Anatomy and Experimental Morphology, University Cancer Center Hamburg, University Medical Center Hamburg-Eppendorf, Hamburg, Germany; ^5^ Department of Oncology, Hematology and Bone Marrow Transplantation with Section Pneumology, Hubertus Wald University Cancer Center, University Medical Center Hamburg-Eppendorf, Hamburg, Germany; ^6^ Department of General, Visceral and Thoracic Surgery, University Medical Center Hamburg-Eppendorf, Hamburg, Germany; ^7^ DFG Emmy Noether Group ‘Neuronal Protein Transport’, Center for Molecular Neurobiology, ZMNH, University Medical Center Hamburg-Eppendorf, Hamburg, Germany

**Keywords:** fascin, metastasis, actin, microtubule, cytoskeletal dynamics

## Abstract

High expression of the actin bundling protein Fascin increases the malignancy of tumor cells. Here we show that fascin expression is up-regulated in more malignant sub-cell lines of MDA-MB-231 cells as compared to parental cells. Since also parental MDA-MB-231 cells exhibit high fascin levels, increased fascin expression was termed as “hyperexpression”. To examine the effect of fascin hyperexpression, fascin was hyperexpressed in parental MDA-MB-231 cells and metastasis was analyzed in NOD scid gamma (NSG) mice. In addition, the effect of fascin mutants with inactive or constitutively active actin bundling activity was examined. Unexpectedly, we found that hyperexpression of both, wildtype (wt) and mutant fascin strongly increased metastasis *in vivo*, showing that the effect of fascin hyperexpression did not depend on its actin bundling activity. Cellular assays revealed that hyperexpression of wt and mutant fascin increased adhesion of MDA-MB-231 cells while transmigration and proliferation were not affected. Since it has been shown that fascin controls adhesion by directly interacting with *microtubules (*MTs), we analyzed if fascin hyperexpression affects MT dynamics. We found that at high concentrations fascin significantly increased MT dynamics in cells and in cell-free approaches. In summary our data show that strong expression of fascin in breast cancer cells increases metastasis independent of its actin bundling activity. Thus, it seems that the mechanism of fascin-stimulated metastasis depends on its concentration.

## INTRODUCTION

Proteins regulating the cytoskeleton are very promising targets for therapy because they are essential to regulate morphological changes associated with tumor cell metastasis. Expression of the actin bundling protein fascin is up-regulated in many tumor types (reviewed in [1]) and it has been shown that this up-regulated fascin expression is associated with increased risk of mortality in breast, colorectal and gastric cancer [2]. Therefore, fascin is discussed as biomarker and cellular target for therapy of malignant cancers. Different groups [3, 4] revealed that direct inhibition of the actin bundling activity of fascin in malignant breast cancer cells had the same metastasis-suppressing effect as its down-regulation. Thus, it seems that the actin bundling activity of fascin accounts for its metastasis-promoting activity. The actin bundling activity of fascin is essential for the formation of filopodia [5] and is also needed for actin stabilization in invadopodia [6], indicating that fascin promotes metastasis by stabilizing cellular protrusions essential for adhesion and invasion.

The fascin molecule is composed of four β-trefoils of which trefoils one and three have actin-binding capacity [7]. The actin binding site in β-trefoil one is inhibited by protein kinase C (PKC)-mediated phosphorylation at Ser-39 [8] resulting in inhibition of actin bundling activity. Obviously, transient activation and inactivation of fascin's actin bundling activity is required for its metastasis-promoting function since overexpression of a phosphomimic and a dephosphomimic mutant did not, while overexpression of wt fascin did increase metastasis of colon cancer cells [9]. In addition to its actin bundling activity, fascin also has actin bundling-independent effects. Villari et al. (2015) [10] showed that fascin can alter microtubule (MT) dynamics by directly binding to MTs via amino acids 234-250. Furthermore, they demonstrated that fascin-mediated regulation of MT dynamics is essential for focal adhesion assembly of breast cancer cells.

In this study we demonstrate that fascin expression is up-regulated in more malignant subtypes of breast cancer MDA-MB-231 cells and reveal that this “fascin hyperexpression” increases metastasis by an actin bundling-independent mechanism.

## RESULTS

### Expression of actin-associated proteins in highly malignant MDA-MB-231 subtypes

**Figure 1:**
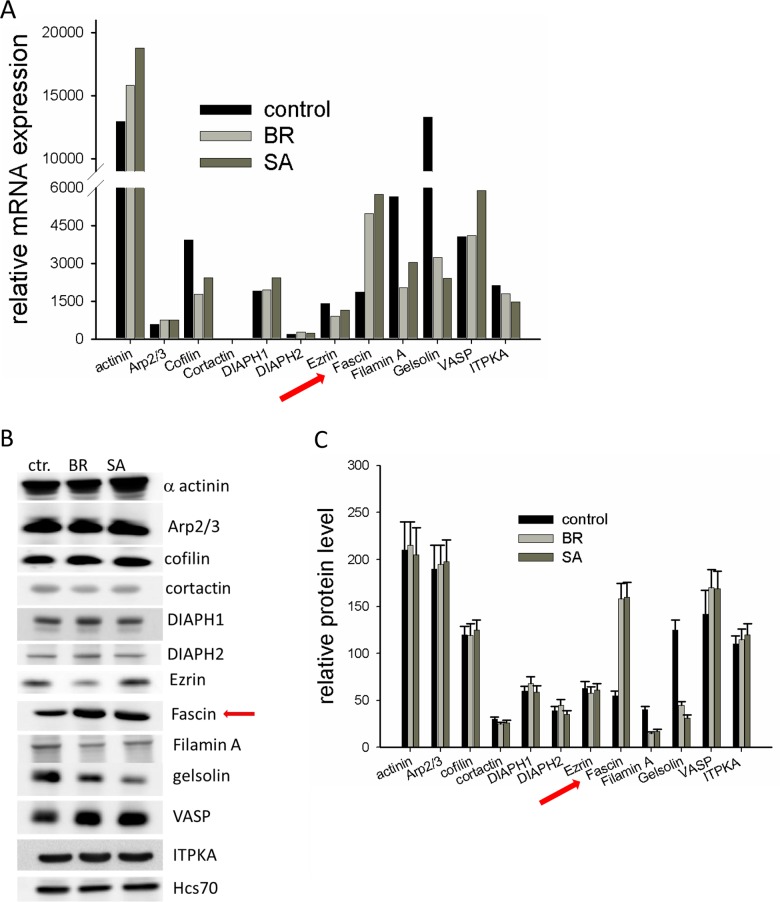
Expression of cytoskeleton-associated proteins in malignant MDA-MB-231 cells **(A)** mRNA expression of different cytoskeleton associated proteins of parental MDA-MB-231, MDA-MB-231-SA and MDA-MB-231-BR was examined by a cDNA expression array (*Affymetrix*® GeneChip) **(B)** Protein lysates of MDA-MB-231, MDA-MB-231-SA and MDA-MB-231-BR were examined for expression of cytoskeleton-associated proteins by western blotting. Shown is one out of three representative western blots. **(C)** Band intensities (n=3) were determined, normalized to Hsc70 loading control and mean ± SD was calculated. Shown are protein levels of actin binding proteins relative to the level of Hsc70.

By serial passaging of the MDA-MB-231 cells in mice by intracardiac injection and subsequent harvest of disseminated cell deposits, different subtypes of cell lines were generated that preferentially disseminate to either the brain (MDA-MB-231-BR) or the bone (MDA-MB-231-SA) [11, 12]. Since cytoskeletal arrangements are required for metastatic spread, we analyzed whether mRNA levels of actin-associated proteins are altered in MDA-MB-231-BR and MDA-MB-231-SA cells as compared to the parental cell line. cDNA microarray data showed that the level of most actin-associated proteins were similar between parental and MDA-MB-231-BR and MDA-MB-231-SA cells. However, mRNA levels of the actin severing proteins gelsolin and cofilin, as well as that of the actin-cross-linking protein Filamin A, were drastically reduced in MDA-MB-231-BR and MDA-MB-231-SA cells as compared to parental MDA-MB-231 cells. Only expression of the actin bundling protein fascin was substantially increased (3-fold, Figure 1A). To confirm these results, protein levels were analyzed by Western blotting (Figure 1B, 1C). The result of this analysis confirmed reduced expression of gelsolin and Filamin A as well as increased expression of fascin in MDA-MB-231-BR and MDA-MB-231-SA as compared to parental cells, while at the protein level cofilin was not altered (Figure 1B, 1C). It has been already shown that gelsolin expression is down-regulated in malignant breast cancer by epigenetic regulation [13]. Whether up-regulated fascin expression and downregulated expression of gelsolin and Filamin-A are related is not known.

Here, we focused on the effect of fascin-upregulation on malignancy of MDA-MB-231 cells. Since at least two different authors revealed that the high basal fascin level is essential for metastasis of parental MDA-MB-231 cells [3, 4], it was interesting to know if a further increase of fascin expression (which we state as “hyperexpression”) further increases malignancy of MDA-MB-231cells.

### Clinical relevance of fascin hyperexpression

**Figure 2:**
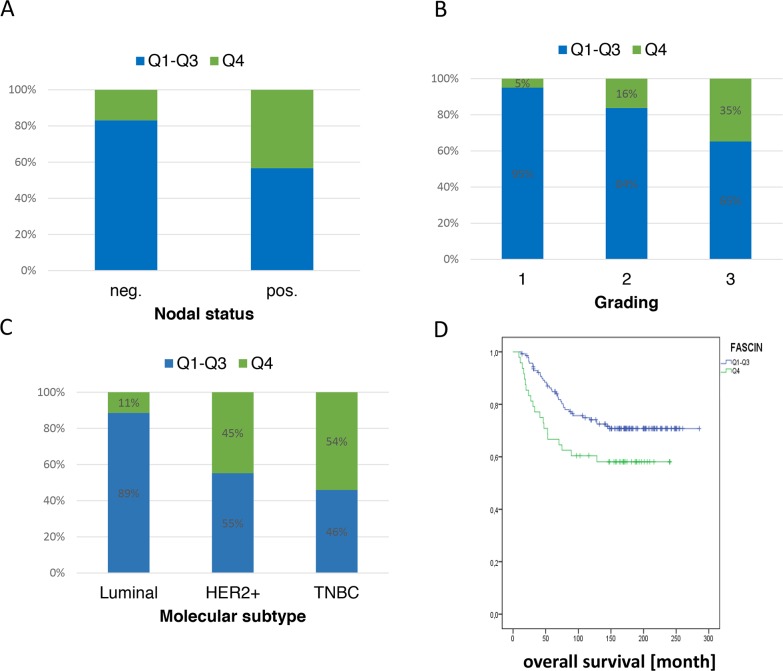
Hyperexpression of fascin is associated with poor clinical outcome Fascin mRNA was analyzed from 194 breast cancer patient samples and correlation analysis with fascin expression, **(A)** nodal status, **(B)** tumor grade, **(C)** molecular subtype and **(D)** overall survival were performed.

It is well documented that a high fascin expression in breast cancer correlates with bad clinical outcome (reviewed in [1]). However it was not clear if a further increase of fascin expression is associated with higher malignancy in breast cancer. Therefore, we analysed fascin mRNA levels in primary breast cancer tissues and grouped the cohort (n=194) into quartiles of which the last one (Q4) represents samples with fascin hyperexpression. To perform correlation analysis with clinical parameter Q1-Q3 (low, moderate-low, moderate-high fascin expression) was compared with Q4 (fascin high expression). This analysis revealed that high fascin mRNA levels significantly correlated with increased lymph node metastasis (p<0.001; Figure 2A) and higher grading (p=0.002; Figure 2B). In addition a significant correlation of fascin hyperexpression with the molecular subtype was found (p<0.001; Figure 2C). Here, HER2+ and triple negative breast cancer (TNBC) tumors were characterized by significantly higher fascin levels. Further, high tumour fascin levels were significantly associated with shorter overall survival (p=0.04; Figure 2D) and showed a trend regarding disease-free survival (p=0.1; Supplementary Figure 1A) in our collective. Interestingly, stratified analyses within the different molecular subtypes showed a significant correlation of high fascin levels with disease free survival in HER2+ and TNBC (p=0.035; Supplementary Figure 1B), whereas no correlation could be found in luminal tumors (p=0.078; Supplementary Figure 1C). These data clearly show that fascin hyperexpression correlates with bad clinical outcome, especially in the more aggressive molecular subtypes of HER2+ and triple negative breast cancer.

### Effect of fascin hyperexpression on actin dynamics *in vitro* and in cells

Our data above show fascin hyperexpression in both, malignant tumor samples from breast cancer patients and in MDA-MB-231 sublines with increased malignancy. It is described that the actin bundling activity of fascin accounts for its malignancy-promoting effect [3, 4]. Therefore, we next analyzed whether fascin's actin bundling activity increases upon fascin hyperexpression. Since the effect of actin-binding-proteins depends on the actin to actin-binding-protein ratio, the actin-to-fascin ratios in parental and in MDA-MB-231-SA cells were determined by western blotting. This analysis revealed an actin-to-fascin ratio of 3:1 in MDA-MB-231 cells and 1:1 in MDA-MB-231-SA cells (Supplementary Figure 2). This result is in line with our data showing a 3-fold higher fascin level in MDA-MB-231-SA cells as compared the parental cell line (Figure 1B, 1C).

**Figure 3:**
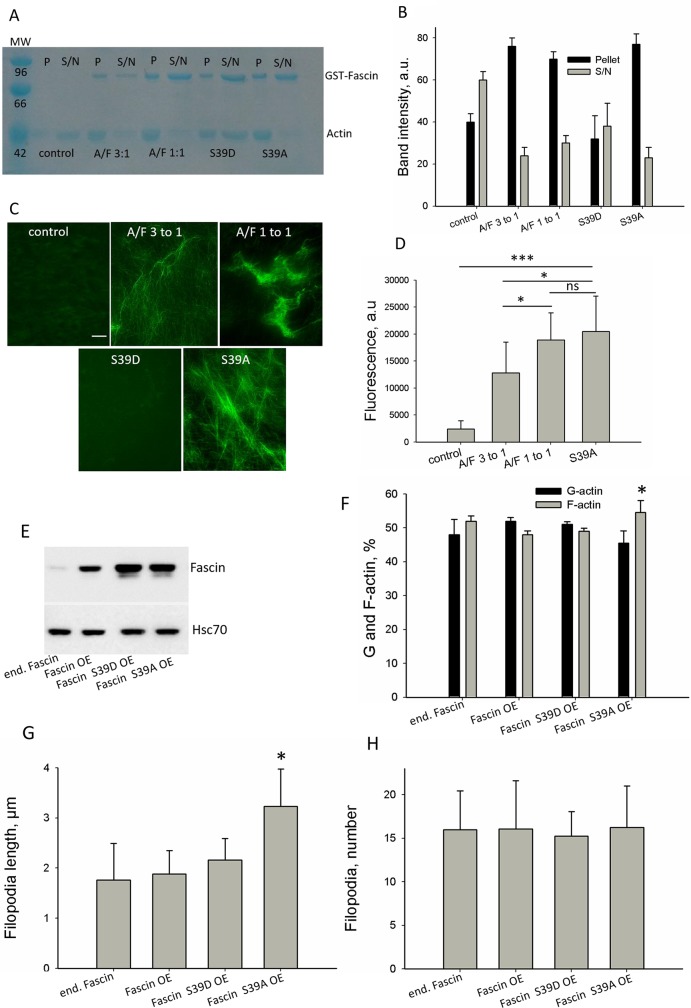
Effect of fascin on actin dynamics in a cell-free system and in cells To analyze the effect of fascin hyperexpression on actin dynamics in cell-free systems, fascin was employed at actin to fascin ratios as occurring in parental MDA-MB-231 (A/F 3:1) or in the more malignant sub-cell line MDA-MB-231-SA (A/F 1:1). **(A)** Fascin or fascin mutants were incubated with F-actin and F-actin bundles were pulled down (P), while F-actin remained in the supernatant (S/N). GST-Fascin bound to F-actin is in the pellet fraction and non-bound GST-Fascin remained in the supernatant. **(B)** Band intensities of actin (42kDa) (n=3) were determined and mean ± SD was calculated. **(C)** Actin alone (control) or in presence of fascin or fascin mutants was stained with Alexa-fluor^®^488-conjugated phalloidin and actin filaments or actin bundles were analyzed by fluorescence microscopy. Bar: 5 μm. **(D)** Fluorescence intensity of actin bundles were analyzed by ImageJ. Shown are mean values of ten different micrographs from two independent experiments. ^*^p<0.05; ^***^p<0.0001. **(E)** MDA-MB-231 cells were lentiviral-transduced with vectors encoding for fascin or fascin mutants. Two weeks after puromycin selection, expression of fascin was analyzed by western-blotting, Hsc70 signals served as loading control. **(F)** Protein lysates of MDA-MB-231 control, fascin or fascin-mutant overexpressing cells were fractioned by centrifugation. F-actin content of pellet and supernatant was analyzed using ITPKA, which is constitutively bound to F-actin, as marker. Then, band intensity was quantified and plotted in graph. Shown are mean values ± SD of three independent experiments.^*^p<0.05. **(G, H)** MDA-MB-231 control, fascin or fascin-mutant hyperexpressing cells were stained for the filopodia marker vasodilator stimulated phosphoprotein (VASP) (see Supplementary Figure 3B) and filopodia length (G) and number of filopodia per cell (H) were measured using the Keyence software. Shown are mean values ± SD of 40 cells from two different experiments.

To analyze if in principle fascin can increase actin bundling at an actin-to-fascin ratio of 1:1 as compared to 3:1, cell-free *in vitro* analysis were performed. In addition, mutants with constitutive active actin bundling activity (S39A) or with impaired actin bundling activity (S39D) were included as controls at actin-to-fascin ratios of 1:1. Determination of actin bundling activity by pulling down actin bundles did not reveal differences between actin-to-fascin ratios of 3:1 and 1:1, but confirmed that the fascin mutant S39D did not exhibit actin bundling activity (Figure 3A, 3B; actin band). In addition, Figure 3A (GST-Fascin band) shows that at an actin to fascin ratio of 2:1 binding of fascin to actin is saturated. However, visualization of actin bundles by fluorescence microscopy clearly showed that at an actin-to-fascin ratio of 1:1 actin bundles were more compact than at a ratio of 3:1 (Figure 3C) and resembled those actin bundles produces by fascin S39A. As expected, the fascin mutant S39D did not induce formation of actin bundles. Quantification of fluorescence signals derived from actin bundles confirmed that the density of actin bundles was significantly increased at an actin-to-fascin ratio of 1:1 as compared to 3:1 (Figure 3D). No differences were found between fascin and the fascin mutant S39A, because in a cell-free system no phosphorylation of fascin occurs. Thus, we conclude that in a cell-free system actin bundling activity of fascin increases at very high concentrations.

Next, we analyzed if this is also true in cells. Therefore, fascin or fascin mutants with inactive or constitutive active actin bundling activity (phosphomimic S39D or dephophosmimic S36A) were stably hyperexpressed in MDA-MB-231 cells using a lentiviral approach. After selection of positive cells with puromycin, hyperexpression of fascin was controlled by western-blot analysis (Figure 3E). Since bundling of actin filaments stabilizes F-actin and is involved in elongation of filopodia, the G-actin to F-actin ratio and filopodia length were analyzed in control cells and in cells hyperexpressing fascin or fascin mutants. To control that hyperexpressed fascin still locates in filopodia, MDA-MB-231 fascin hyperexpressing and control cells were stained with an anti-fascin antibody. This analysis revealed that hyperexpressed wt fascin accumulated at the tip and throughout the filopodia while fascin S39D was located diffusely in lamellipodia-like structures and Fascin S39A at the tip of filopodia (Supplementary Figure 3A).

Analysis of the G- to F-actin ratio and filopodia length revealed that only hyperexpression of the constitutive active fascin mutant (S39A) increased the F-actin concentration (Figure 3F) as well the length of filopodia (Figure 3G and Supplementary Figure 3B). The number of filopodia was not altered after hyperexpression of fascin or fascin mutants (Figure 3H). We assume that accumulation of high concentrations of constitutive active fascin at the leading edge of MDA-MB-231 cells increases actin bundling and thus stimulates elongation of filopodia. However, overexpression of wt or constitutive inactive fascin (S39D) had no effect on F-actin concentration and on filopodia elongation. This result shows that fascin S39D does not act as dominant negative mutant, most likely because it is not able to replace endogenous fascin from the tips of filopodia, because of its diffuse localization in lamellipodia (see Supplementary Figure 3A). In addition the result reveals that overexpression of wt fascin does not increase the F-actin concentration and elongation of filopodia, indicating that hyperexpression of wt fascin does not further stimulate actin bundling in cells.

In conclusion, our results indicate that only hyperexpression of a constitutive active fascin mutant increases actin bundling while hyperexpression of wt fascin has no effect.

### Hyperexpression of fascin in MDA-MB-231 cells increases metastasis independent of its actin-bundling activity

Our data above show that fascin hyperexpression does not further increase the formation of actin bundles in cells, indicating that the effect of fascin on actin bundling is saturated at a certain fascin level. These data assume that the effect of fascin hyperexpression on malignancy of breast cancer (see Figure 2) does not result from its actin bundling activity.

**Figure 4:**
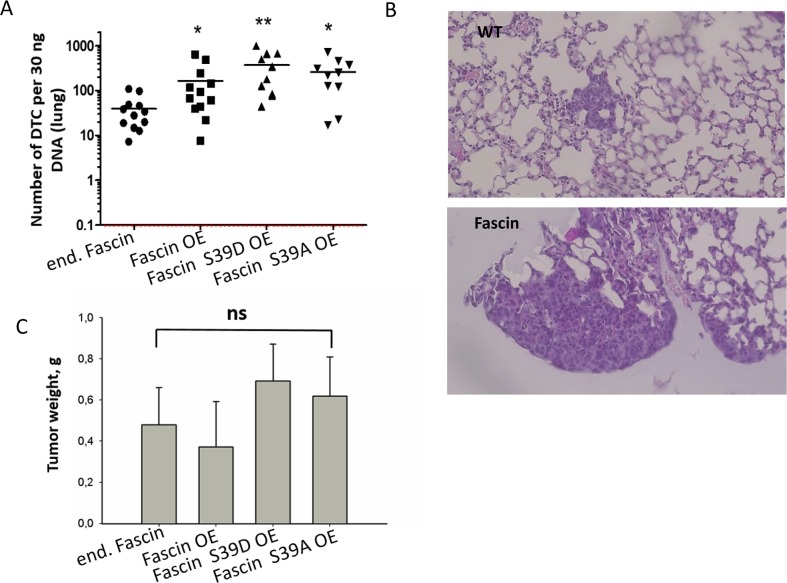
Fascin hyperexpression increases metastasis independently of its actin bundling activity MDA-MB-231 control, fascin or fascin-mutant overexpressing cells were injected into the flank of NSG mice; 10 mice per cell line were used. About 6 weeks later the number of cells in the lung was analyzed by **(A)** *Alu* qRT-PCR and by **(B)** histological examination of HE-stained lungs. Please note the logarithmic scale of the y-axis in (A). ^*^p<0.05, ^**^p<0.001 (Mann Whitney test). DTC: Disseminated tumor cells. Bar: 20 μm. (B) Mean values of tumor weight in gram (g) ± SD. **(C)** Primary tumors were dissected and weighed. Shown are mean values ± SD from ten to twelve tumor per group.

In order to validate this assumption, metastasis of MDA-MB-231 cells hyperexpressing fascin, fascin-S39D or fascin-S39A, as well as parental vector-transfected MDA-MB-231 cells (control), were analyzed in immunodeficient mice. The cells were injected subcutaneously into the flank of the mice and the animals were sacrificed when the primary tumor had reached a size of about 1.5 cm^2^ (24 days after injection). To control that fascin expression did not decrease during growth of MDA-MB-231 cells in mice, the primary tumors were embedded in paraffin and stained for fascin. Analysis of the samples revealed that fascin expression remained stable after tumor growth in mice (Supplementary Figure 4). Spontaneous metastasis of the cells was analyzed by *Alu*-qRT-PCR and by histology (for details, see methods). We found that fascin-hyperexpression caused a 4-fold increase in the metastatic cell burden in the lungs as determined by *Alu* qRT-PCR (*p*=0.022, Mann Whitney test) as compared to control cells. Analysis of lung metastasis by histology revealed that both, the number and size of lung metastases were increased in fascin-hyperexpressing relative to control MDA-MB-231 cells (Figure 4B). Importantly, fascin hyperexpression did not alter primary tumor growth in our xenograft model (Figure 4C). Thus, fascin hyperexpression increases metastasis, but not tumor growth.

The metastatic cell loaded in the lungs of mice injected with MDA-MB-231 cells expressing the fascin mutant with inactive actin bundling activity (S39D) was even 9-fold higher as compared to control cells (*p*=0.0007, Mann Whitney test, Figure 4A), while the number of cells expressing the constitutive active form (S39A) of fascin was 6.5-fold higher (*p*=0.004, Mann Whitney test, Figure 4A). However, the higher effect of fascin mutants on metastasis relative to wt fascin could be explained by the fact, that the level of fascin mutants in MDA-MB-231 cells is higher than that of wt fascin (see Figure 3E).

In summary these *in vivo* data confirm our assumption that fascin hyperexpression increases metastasis independent of its actin bundling activity.

### Cellular processes accounting for fascin-hyperexpression-stimulated metastasis

**Figure 5:**
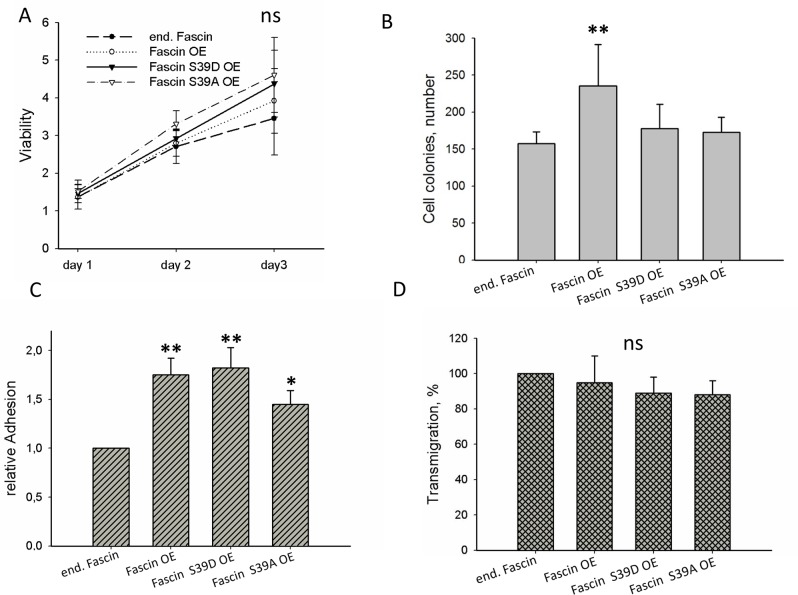
Cellular effects of fascin hyperexpression **(A)** Viability was measured by the MTT-assay. Values were normalized to adhesion controls of cells incubated for 16 h **(B)** For colony assay 500 cells were seeded into 6-well plates and after incubation for 10 days colonies were stained with Giemsa and counted. **(C)** Adhesion was measured by determining the number of cells adhering to the Petri dish. **(D)** Transmigration was analyzed by the Transwell assay. In parallel cellular adhesion was measured and transmigrated cells were normalized to adherent cells. Control cells were set to 100%. Shown are mean values ± SD of at least three independent experiments. ^**^p<0.001, ^*^p<0.05.

In order to analyze which cellular processes drives fascin-hyperexpression-stimulated metastasis, viability (Figure 5A), colony formation (Figure 5B), adhesion (Figure 5C) and trans-migration (Figure 5D) were compared between control and fascin or fascin-mutant hyperexpressing MDA-MB-231 cells. This analysis revealed that hyperexpression of fascin significantly increased colony formation and adhesion of MDA-MB-231 cells but had no significant effect on viability and on transmigration. In the case of adhesion, MDA-MB-231 cells hyperexpressing fascin, fascin S39D and fascin S39A significantly increased adhesion as compared to control cells (p=0.002, 0.0046, 0.018). However, colony formation was only significantly increased by hyperexpressing wt fascin (p=0.03). From this result we conclude that fascin hyperexpression only increases colony formation when its actin bundling activity can be transiently regulated by phosphorylation. Thus, the effect of fascin on colony formation seems to be dependent on its actin bundling activity. The stimulating effect of fascin hyperexpression on adhesion, however, seems to be independent of its actin bundling activity because hyperexpression of fascin as well as hyperexpression of fascin mutants with constitute active (S39A) or inactive (S39D) actin bundling activity had similar stimulated effects on adhesion of MDA-MB-231 cells.

### Fascin hyperexpression increases microtubules dynamics

**Figure 6:**
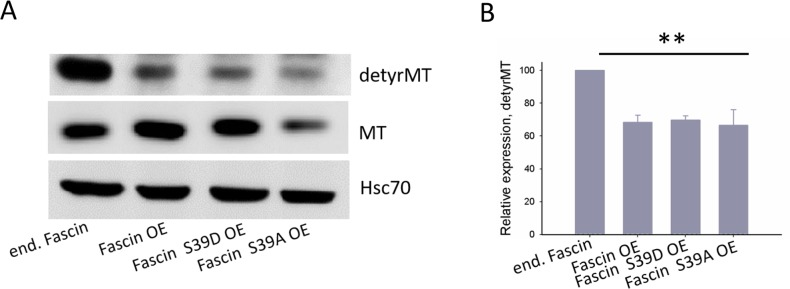
Fascin hyperexpression destabilizes MTs **(A)** Protein lysates of MDA-MB-231 control, fascin or fascin-mutant hyperexpressing cells were blotted against detyrosinated (detyrMT), thus stabilized MTs. **(B)** Band intensities of detyrMT (n=3) were determined, normalized to MT-signals (which had been normalized to Hsc70 signals before) and mean ± SD was calculated. ^**^p<0. 001. Values of control cells were set to 100%.

Our data above show that fascin-hyperexpression in MDA-MB-231 cells increases metastasis *in vivo* and adhesion *in vitro* independent of its actin bundling activity. A recent study revealed that depletion of fascin in MDA-MB-231 cells resulted in less dynamic MTs. In addition the authors showed that fascin directly binds to MTs thereby regulating cellular adhesion [10]. It was thus interesting to analyze if hyperexpression of fascin in MDA-MB-231 cells also alter MT dynamics. For this purpose, control and fascin hyperexpressing MDA-MB-231 cells as well as cells hyperexpressing the inactive or constitutively active actin bundling fascin mutant were analyzed for expression of detyrosinated (thus stabilized [14]) MTs by western blotting. As shown in Figure 6, hyperexpression of wt and mutant fascin reduced the level of detyrosinated MTs by about 40%. Thus, hyperexpression of fascin seems to increase MT dynamics.

### High fascin concentration alters MT-dynamics in a cell-free system

In order to analyze the mechanism by which fascin hyperexpression regulates MT dynamics, cell-free *in vitro* experiments were performed employing MT-to-fascin ratios as occurring in fascin overexpressing cells (1:1, see Supplementary Figure 2).

**Figure 7:**
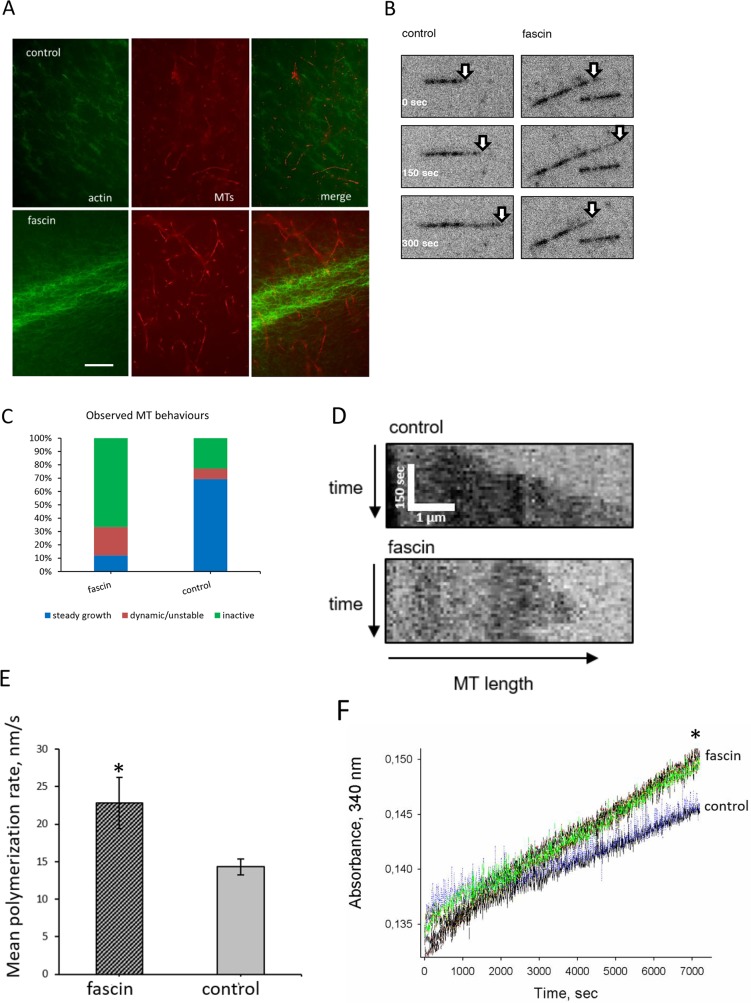
Fascin regulates MT-dynamics *in vitro* **(A)** First Alexa-fluor^®^488-coupled phalloidin-stained actin was applied to chamber slides. After 20 min of incubation, rhodamine labeled taxol-stabilized MTs were added and incubation was continued for another 15 min. Images were taken by fluorescence microscopy. The last image show merges of F-actin (green) and MTs (red). Bar: 5 μm. **(B-C)** Show TIRF-analysis of fascin-controlled MT dynamics; (B) representative micrographs, (C) quantitative analysis of MT-dynamics, **(D)** kymographs of MT dynamics in presence or absence of fascin, **(E)** polymerization rates of MTs. Shown are mean values of three measurements ± SEM **(F)** For confirmation, polymerization of non-labeled, non-taxol stabilized MTs was again measured at 340 nm in the presence or absence of fascin for 2 hours. Shown are three measurement of each sample.

In principle, it is possible that actin binding of fascin influences its effect on MT dynamics. In this case fascin should connect the actin cytoskeleton with MTs. To analyze this, Alexa-fluor^®^488 labeled actin and taxol-stabilized, rhodamine labeled MTs with or without fascin were incubated and actin filaments and MTs were visualized by and fluorescence microscopy. As positive control Alexa-fluor^®^488 labeled actin and taxol-stabilized MTs were incubated with DIAPH2 which binds actin with its FH1/FH2 domains as well as MTs with its FH2 domain [15]. DIAPH2 induced the formation of polarized actin filaments and recruited MTs to F-actin (Supplementary Figure 5). However, in presence of fascin no co-localization between F-actin and MTs were detected (Figure 7A). Thus, it is unlikely that actin binding of fascin influences its effect on MT-dynamics.

Since our data above revealed that in MDA-MB-231 cells fascin hyperexpression increases MT dynamics we next analyzed if this is mediated directly or indirectly via cellular fascin-interaction partners. For this purpose growth of HiLyte Fluor™-647 labeled, non-taxol-stabilized MTs were analyzed by TIRF microscopy. Indeed, this analysis revealed that in the presence of fascin the percentage of both growth and shrinking of MTs (“dynamic MTs”) was increased as compared to control (Figure 7B, 7C). In addition quantitative evaluation of kymographs showed that fascin significantly (P=0.007) accelerated MT-polymerization (Figure 7D, 7E). This result was confirmed by measuring polymerization of non-labeled and non-taxol-stabilized MTs in a Tecan reader at 340 nm, showing that fascin significantly (P=0.0019) increased the velocity (by 25%) of MT polymerization (Figure 7F). In conclusion, our data show that at high concentrations fascin increases MT-dynamics.

## DISCUSSION

Proteins regulating the cytoskeleton are promising targets for therapy of malignant cancers (reviewed in [16]). In this study, we screened for actin-associated proteins which are differentially expressed in more malignant sub-cell lines of MDA-MB-231 (MDA-MB-231-SA and MDA-MB-231-BR). Thereby, we found that fascin was the only actin-associated protein examined whose expression was up-regulated in both MDA-MB-231-SA and MDA-MB-231-BR cells. It has been described by different groups that high fascin expression increases malignancy of different tumor types (reviewed in [2]). However, parental MDA-MB-231 cells already expressed high fascin levels and it has been shown that in these cells fascin is essential for metastasis in SCID mice [3]. Thus, it was not clear if a further increase of fascin expression, as observed in MDA-MB-231-SA and MDA-MB-231-BR cells, further increases malignancy. To analyze this question, samples from breast cancer patients were analyzed for fascin expression. Groups containing patients with low, moderate-low and moderate-high fascin expression levels showed similar overall and progression-free survival times while the group with very high fascin expression (fascin hyperexpression) showed significant decreased survival. Thus it seemed that fascin hyperexpression increases malignancy of breast cancer cells.

To analyze the mechanism behind this, we compared fascin's actin bundling activity at actin-to-fascin ratios as occurring in parental MDA-MB-231 (3:1) and in MDA-MB-231-SA (1:1) cells. In addition, mutants with constitutively active or inactive actin bundling activity were analyzed. In cell-free assays actin bundling activity of fascin was increasable at an actin to fascin ratio of 1:1 as compared to 1:3. However, in cells fascin hyperexpression did not further enhance the effect of fascin on F-actin, indicating that the effect of fascin on actin dynamics is saturated at a certain fascin level. This result implied that the effect of fascin hyperexpression on malignancy of breast cancer cells did not result from its actin bundling activity. To analyze this assumption, metastasis of MDA-MB-231 cells hyperexpressing fascin or fascin mutants with inactive or constitutive active actin bundling activity was examined. Indeed, we found that all fascin variants strongly increased metastasis, showing that fascin hyperexpression further stimulates malignancy independent of its actin bundling activity. Cellular assays revealed that only adhesion was enhanced after overexpression of fascin and fascin mutants, indicating that fascin hyperexpression stimulates metastasis by promoting cellular adhesion.

Villary et al. (2015) [10] demonstrated that fascin is essential for focal adhesion assembly and revealed that for this process binding of fascin to MTs was required. They found that depletion of fascin in MDA-MB-231 cells resulted in less dynamic MTs with reduced growth rates and proposed a model where dynamic MTs are important for focal adhesion assembly. Since these findings indicate that the actin bundling independent effect of fascin hyperexpression on cellular adhesion could be mediated by MT-regulation, we examined if fascin hyperexpression alters MT dynamics and found that hyperexpression of fascin increased MT dynamics in MDA-MB-231 cells. In addition, cell-free *in vitro* experiments, where we employed MT-to-fascin ratios as occurring in fascin hyperexpressing cells, revealed that at high concentrations fascin increases MT dynamics. Furthermore, we found that fascin does not connect the actin cytoskeleton with MTs, supporting the idea that in cells fascin can only bind to MTs when actin binding by β-trefoil-3 is blocked through phosphorylation of S274 [10].

In conclusion, our data show that the stimulating effect of fascin hyperexpression on metastasis is not dependent on its actin bundling activity. Since we found that fascin hyperexpression increased MT-dynamics, it is tempting to speculate that fascin-mediated regulation of MTs and fascin-stimulated metastasis are related. However, future experiments are necessary to prove this assumption. In addition, it is interesting to analyze which cellular processes are controlled by fascin-mediated MT-regulation since MTs regulate multiple cellular processes, including cell division, migration, transport and invasion, all of which can trigger metastasis [17, 18]. Furthermore, it should be examined if fascin-controlled MT-dynamics mediate the protecting effect of fascin against the MT-targeting drug Taxotere [19].

**Figure 8:**
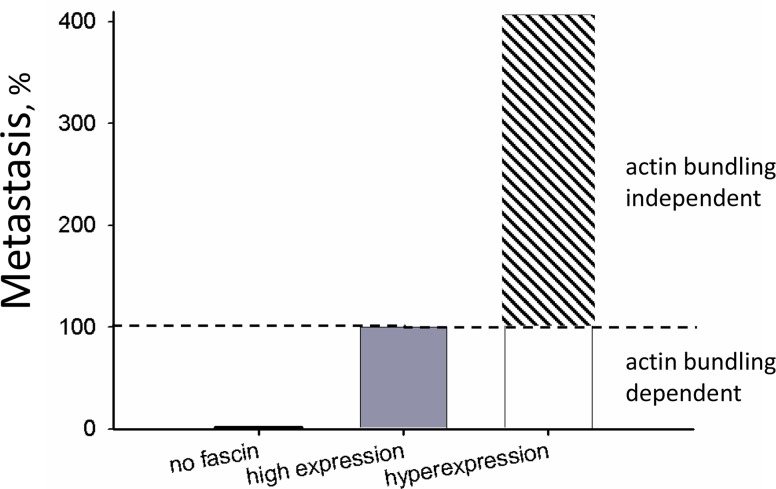
Actin bundling-independent effect of fascin on metastasis The number ofcontrolMDA-MB-231 cells that had metastasized to the lung were set to 100% (“high expression”). For comparison, data from fascin-depleted cells (“no fascin”) were taken from Chen et al., 2010 [3] who analyzed the effect on fascin-down regulation on metastasis of MDA-MB-231 cells. Data from fascin hyperexpression are depicted in Figure 4A.

To summing up, our data demonstrate that fascin expression increases with malignant progression of breast cancer cells and reveal that this increased fascin expression further enhances metastasis independent of its actin bundling activity (see Graph in Figure 8). This conclusion should be taken into account for development of drugs inhibiting fascin activity.

## MATERIALS AND METHODS

### Analysis of mRNA expression data in cell lines and breast cancer tumor tissue samples

Microarray data was analyzed from pooled triplicate experiments of the three different subclones of the MDA-MB-231 cell line (parental, SA and BR) as described [20]. Array data is available at http://www.ncbi.nlm.nih.gov/geo GSE44354. Authentications of all cell lines were conducted by short tandem repeat (STR) profiling to exclude cross-contamination between the cell lines.

Fascin mRNA levels was furthermore analysed using microarray data (Affymetrix HG-U133A) from a cohort of 194 mammary carcinoma enrolled in the Department of Gynecology, Hamburg University Medical Center. Cohort characteristics as well as technical details have been previously described [21]. Informed consent for the scientific use of tissue materials, which was approved by the local ethics committee (Ethik-Kommission der Ärztekammer Hamburg, #OB/V/03), was obtained from all patients. The study was performed in accordance to the principles of the declaration of Helsinki and REMARK criteria (Mcshane LM, Altman DG, Sauerbrei W, Taube SE, Gion M, Clark GM. REporting recommendations for tumor MARKer prognostic studies (REMARK) 2005;2:416–422).

According to the fascin values (probe set 210933s_at) the cohort was divided into quartiles of similar size, representing low, moderate-low, moderate-high and high expression of the analyzed factor. Correlations between fascin levels and clinicopathological factors such as histological grading (for details, see http://pathology.jhu.edu/breast/grade.php), stage, lymph node involvement, estrogen and progesterone receptor status (ER, PR) and molecular subtypes, were statistically examined by χ2-tests. Overall survival was analysed by Kaplan-Meier analysis and Log-Rank-Tests. Probability values less than 0.05 were regarded as statistically significant. All statistical analyses were conducted using SPSS software Version 23 (SPSS Inc., Chicago, IL, USA).

### Recombinant expression of fascin in bacteria or in MDA-MB-231 cells

The cDNA of fascin was first cloned into the pGEM®-T Easy vector (Promega, Mannheim, Germany) and the cDNA was mutated by Quick change mutagenesis to S39D or S39A. Then, wt and mutated cDNAs were sub cloned into the vector pGEX-6P-2A and expression in *E-coli* and protein purification were performed as described [22].

For hyperexpression of fascin and fascin mutants in MDA-MB-231 cells, the cDNAs of wt and mutated fascin were cloned into the lentiviral vector LeGo-iC2-Puro+ which was a friendly gift from Kristoffer Riecken. For virus production HEK239-3T cells were used. 10 μg LeGo-iC2-Puro+, 10 μg gagpol packaging plasmid, 5 μg pRSVrev packaging plasmid and 2 μg VSV-G vector (coding for the envelop protein) were added to 750 μl Optimem and incubated for 5 min at RT. In parallel 40 μl Lipofectamine 2000 was mixed with 750 μl Optimem and incubated for 5 min at RT. Both samples were mixed, incubated for 20 min at RT and applied to HEK-293-T cells which had been seeded to 10 cm dishes. HEK-293-T medium containing the virus was transferred to MDA-MB-231 cells grown in 6-well plates after 24 h, 48 h und 72 h. After further incubation for 24 h, the virus-containing medium was changed against puromycin (2 μg/ml) containing DMEM/10% FCS. Overexpression of fascin was analyzed by western blotting after one week. Primary antibodies against fascin (Santa Cruz SC-251) and Hsc70 (Santa Cruz SC-7289) were used in a dilution of 1:000 or 1:5000, respectively. Secondary antibodies against rabbit (Santa Cruz SC-2054) or mouse (Santa Cruz SC-2055) were used in dilutions of 1:5000.

### Determination of actin-to-fascin or MT-to-fascin ratios in cells

To examine the effect of fascin hyperexpression on actin and MT dynamics in a cell-free system it is important to know the actin- (or MT-) to-fascin ratio. For this purpose, we blotted fascin, actin or tubulin standards as well as protein extracts from MDA-MB-231 and MDA-MB-231-SA cells against fascin (antibody from Santa Cruz SC-251, dilution 1:1000), actin (antibody from Sigma A2066, dilution 1:10,000) or tubulin (antibody from Sigma T1026, dilution 1:1000). Band intensities were analyzed by ImageJ. Based on the band intensities of protein standards, concentrations of fascin, actin and tubulin per mg cellular protein were calculated. Experiments were performed at least three times and mean ± SD values were calculated.

### Analysis of the actin structure in a cell-free system

All reagents used for cell-free analysis of actin or MTs dynamics were from tebu-bio Cytoskeleton (Offenbach, Germany).

The effect of fascin on F-actin structure was analyzed by florescence microscopy: Actin (1 mg/ml) was incubated on ice for one hour and was then centrifuged for 15 min at 13.000 g, 4°C. The supernatant was used. 18 μl G-buffer (see [22]) including 200 μM ATP and 1 mM DTT, 2 μl actin, 2.5 μl Alexa-fluor^®^ conjugated phalloidin (1:25 dilution) as well as 2.5 μl 10 x assay buffer (see [22]) were mixed and applied to a poly-L-lysine-covered chamber slide (Ibidi, Martinsried, Germany). After about 20 min, actin bundles or cross links were monitored by fluorescence microscopy. Fluorescence intensity of actin bundles were analyzed by ImageJ. Five areas where actin bundles had been formed were analyzed per micrograph. In addition, five areas without actin bundles were analyzed (background). Then, mean values of fluorescence intensity of actin bundles and background were calculated and fluorescence intensity of actin bundles were subtracted from background. Five micrographs from two independent experiments were analyzed.

Actin bundling was measured by pull down experiments as described [22].

### Determination of F-to-G-actin ratio

MDA-MB-231 cells, grown in 15 cm Petri dishes were washed twice with cold PBS. 3 ml PBS was added to the Petri dish, the cells were scraped with a cell scraper and centrifuged for 5 min at 500 g 4°C. Cell lysis was performed by adding 500 μl lysis buffer (100 mM HEPES, pH7.9; 15 mM MgCl_2_; 100 mM KCl, 1 mM DTT and proteinase inhibitor (Roche)), resuspending the cells and incubating them on ice for 15 min. Thereafter, 10% IGEPAL CA-6630 was added and the sample was vortexed for 10 sec and centrifuged for 30 s at 11.000 g 4°C. In the supernatant G-actin is enriched. The pellet was resuspended again in 70 μl extraction buffer (20 mM HEPES pH 7.9; 1.5 mM MgCl_2_, 0.42 M NaCl, 0.2 mM EDTA, 25% glycerol, 1 mM DTT and proteinase inhibitor (Roche)), incubated on a shaker for 30 min at 4°C und centrifuged for 5 min at 21, 000 g 4°C. In this supernatant F-actin is enriched.

Actin standards and the supernatants (25 μg protein each) were applied to SDS-PAGE and after blotting the gel, antibodies against actin (Sigma A2066 dilution, 1:10,000) and ITPKA (Santa Cruz SC-11206, dilution 1:2000) were applied.

Thereafter, band intensities were determined by ImageJ. Since ITPKA is constitutively bound to F-actin [23] the distribution of F-actin in the supernatants was analyzed by determining the band intensities obtained by the ITPKA antibody. The concentration of actin was analyzed by determining the band intensity obtained by the actin antibody relative to the band intensity of the actin standard. Using these parameters, the actin concentration per 25 μg whole protein extract was analyzed. Finally the percentage of G-actin and F-actin in pellet and supernatant was calculated.

### Immunofluorescence

Cells seeded on chamber slides (Ibid, Munich, Germany) were washed with warm PBS and fixed with warm 4% paraformaldehyde (diluted in DMEM, 10% FCS) at 37°C for 15 min. For permeabilization, the cells were incubated 3-times with warm 0.1% Triton X-100/PBS for 5 min at 37°C and washed then 3-times with PBS for 10 min. The first antibodies (anti-fascin, Santa Cruz sc21746; anti VASP, Santa Cruz sc-46668) were diluted 1:200 in PBS/4% FCS and incubated over night at 4°C. After washing 3-times with PBS for 10 min, the secondary Alexa-fluor^®^488 conjugated antibody (diluted 1:2000 in PBS/4% FCS) was added and the cells were incubated for 1 hour RT in the dark. Finally, the cells were washed 3-times with PBS and analyzed by fluorescence microscopy.

### Measurement of MT-actin interaction and MT dynamics a cell-free system

To analyze interaction of MTs with F-actin. We first produced taxol-stabilized MTs: Labeled MTs and unlabeled MTs were mixed: 37 μl MTs (10 mg/ml), 1 μl rhodamine labeled MTs (10 mg/ml), 0.65 μl DTT (100 mM) 6.5 μl GMPC (non-hydrolysable GTP, 10 mM) 19.85 μl GT-buffer (80 mM PIPES, pH 6.9; 2 mM MgCl_2_; 0.5 mM EGTA) were mixed, frozen to 5 μl aliquots in liquid nitrogen and stored at -80°C. For visualization of MT-formation 1 mM GTP and 20 mM taxol were added to GT-buffer. This buffer was pre-warmed in a water bath at 37°C. The MT-stock (6 mg/ml; see above) were thawed and incubated for 30 min in a water bath at 37°C. 1 μl of this solution was diluted in 360 μl pre-warmed GT-buffer (0.01 mg/ml MTs) containing 200 μM taxol and 1 mM GTP and was incubated over night at room temperature. Afterwards, MTs were for incubated for 30 min in a water bath at 37°C, these MTs were used for analysis of interaction of MTs with F-actin. Therefore, 10 μl of Alexa-fluor^®^488 labeled actin solution (see above) was applied to chamber slides. After about 20 min of incubation, when F-actin had sticked to the bottom of the chamber slide (Ibidi, Martinsried, Germany) 10 μl of the MT solution was added and incubation was continued for about 20 min. F-actin and MTs were monitored by fluorescence microscopy.

For analysis of MT-polymerization unlabeled MTs were used: 10 μl (10 mg/ml) ice cold MTs were thawed and added to 90 μl prewarmed (37°C) GT-buffer/1 mM GTP/10% glycerol. Polymerization was measured in prewarmed (37°C) 96-well plates in a Tecan reader (Infinite 200) at 340 nM/37°C for 2 h.

Analysis of MT dynamics using TIRF microscopy was performed as described previously [24]. Briefly, double cycled MT seeds were produced from unlabeled porcine brain tubulin, tubulin-biotin and tubulin-Hylite647 (all from Cytoskeleton) as described [25]. Flow chambers, assembled from glass coverslips and microscopic slides were functionalized by sequential incubation with 0.2 mg/ml PLL-PEG-biotin (Susos AG, Switzerland), 1 mg/ml NeutrAvidin (*Invitrogen*) and 5 mg/ml κ-casein (Sigma) in MRB80 buffer (80 mM PIPES, 4 mM MgCl2, 1 mM EGTA). MT seeds were immobilized in the imaging flow chamber through biotin-neutravidin interaction. Polymerization was induced by addition of imaging buffer (MRB80 buffer supplemented with 15 μM porcine brain tubulin, 0.5 μM rhodamine-tubulin, 50 mM KCl, 1 mM guanosine triphosphate, 0.2 mg/ml κ-casein, 0.1% methylcellulose, and oxygen scavenger mix [50 mM glucose, 400 μg/ ml glucose oxidase, 200 μg/ml catalase, and 4 mM DTT]) either in the presence or absence of 2 μM fascin. Imaging was done on a Visiscope TIRF/FRAP/PA imaging system. TIRF microscopy based on Nikon Ti-E equipped with a perfect focus system (Nikon), Nikon CFI Apo TIRF 100x, 1.49 N.A. oil objective, a back focal TIRF scanner for suppression of interference fringes (Ilas-2, Roper Scientific France/ PICT-IBiSA, Institut Curie) and controlled with VisiView software. 405, 561, 488 and 647 nm laser lanes were used for illumination and activation of respective fluorophores. Tubulin-Hylite647 fluorescence was collected through an ET 405/488/561/640 Laser Quad Band filter with a The ORCA-Flash4.0 LT sCMOS camera.

### Adhesion assay

Cells were trypsinized, suspended in medium containing 10% FCS and 30,000 cells were seeded to a polylysine-covered 8-chamber slides (Ibidi, Munich). After further incubation for 2 h, the cells were fixed with 4% paraformaldehyde/PBS and stained with 4′,6-Diamidin-2-phenylindol (DAPI). Thereafter, the cell number was determined using the Keyence software function Hybrid Cell Count.

### Transwell invasion assay

50,000 cells were seeded into Boyden chambers as well as in parallel wells to measure adhesion. After incubation for 16 h, the cells seeded to the parallel wells were *trypsinized* and counted by the CASY-cell counter to calculate the number of adhered cells. The cells which had transmigrated through the pores of the membrane were fixed and its number determined as described [23]. For normalization, the ratio of transmigrated/adhered cells were calculated.

### Measurement of cell viability and colony formation

Cell viability was measured by the MTT-Assay as described [26]. For determination of colony formation, 500 cells diluted in 2 ml cell culture medium (DMEM/10% FCS) were applied to a 6-well plate and the cells were incubated for 2 weeks. Thereafter, the cells were washed with PBS and fixed with 4% paraformaldehyde/4% sucrose. After washing with ddH_2_O, cells were stained with 500 μl Giemsa Azur-Eosine-Methylblue solution (1:10) for 10 min. The cells were washed again with ddH_2_O, dried at room temperature and number of colonies were counted.

### Analysis of spontaneous metastasis in a subcutaneous xenograft SCID mouse model

1 × 10^6^ cells were suspended in cell culture medium without supplements and were subcutaneously injected between the scapulae of narcotised (O_2_/CO_2_) mice. Fifteen mice per group were analysed. The mice were sacrificed by cervical dislocation when the first primary tumors had a size of 1.5 cm^2^. Primary tumors were excised, weighted, and processed for histological examinations. The lungs were dissected, embedded in paraffin and stained with hematoxylin/eosin or stored at -20°C for later DNA-extraction. Lung DNA was extracted using the DNeasy blood and tissue kit from Quiagen. Human tumor cells which had metastasized to the lungs were quantified by real-time PCR using established primers specific for human Alu sequences [27]. Numerical data were determined by generating a standard curve from log-fold dilutions of DNA from 1 × 10^6^ cell culture grown MDA-MB-231 cells. Negative controls included lung tissue from non-treated mice. For each sample, analyzes were done in duplicates and as independent at least twice.

## SUPPLEMENTARY MATERIALS FIGURES


