# Supplementary material for: Strong fascin expression promotes metastasis independent of its F-actin bundling activity
Source: Oncotarget. 2017 Nov 1;8(66):110077–91. doi: 10.18632/oncotarget.22249 (PMC5746366; doi:10.18632/oncotarget.22249)
Supplement: Supplementary file 1 [file oncotarget-08-110077-s001.pdf]

Strong fascin expression promotes metastasis independent of its F-actin bundling activity

SUPPLEMENTARY MATERIALS

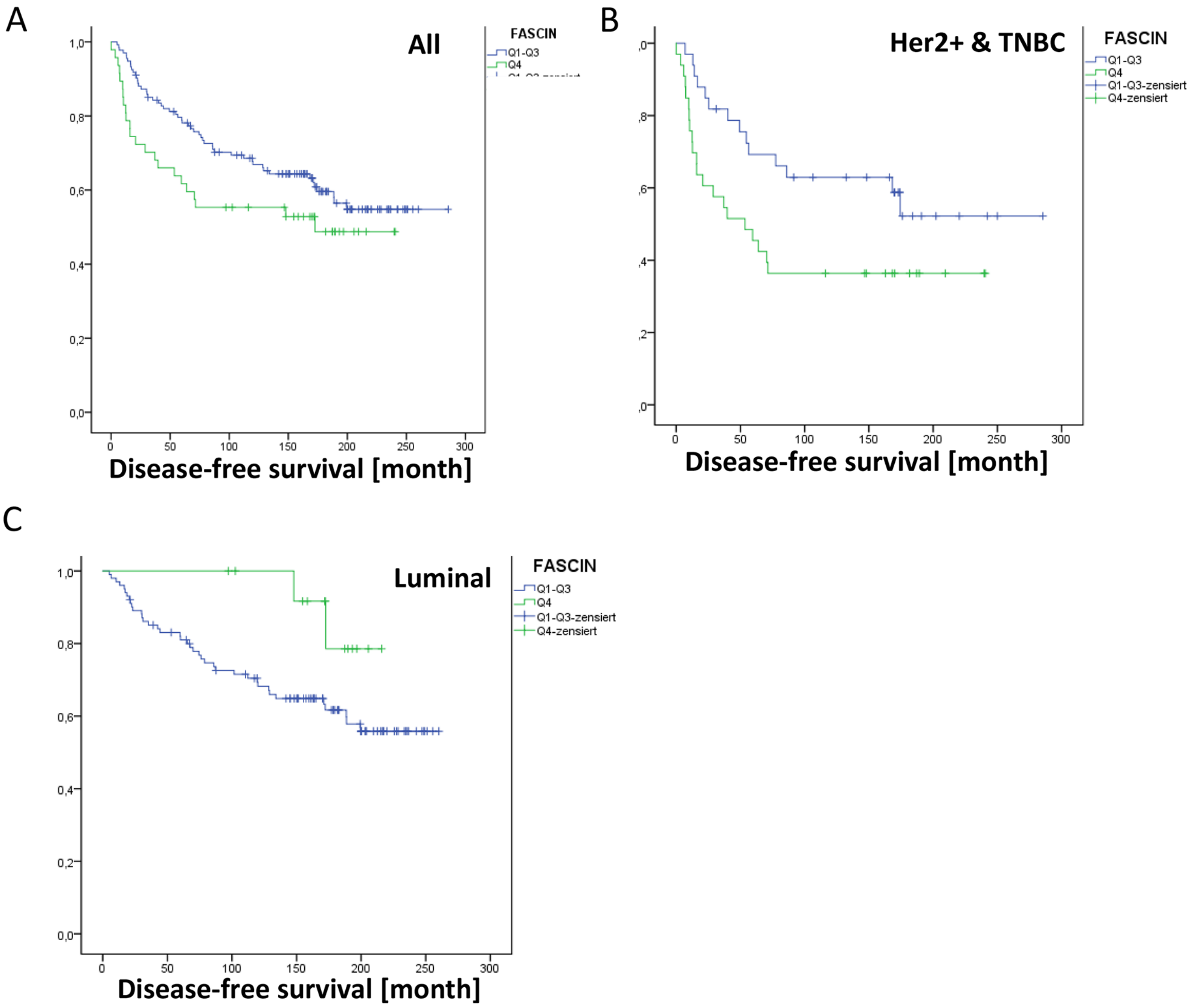

Supplementary Figure 1: Hyperexpression of fascin and disease-free survival. Fascin mRNA was analyzed from 194 breast cancer patient samples **(A)**, “All”). The samples were classified in Her2+, TNBC **(B)** and luminal **(C)** subtypes and correlation analysis with fascin expression and disease-free survival was performed.

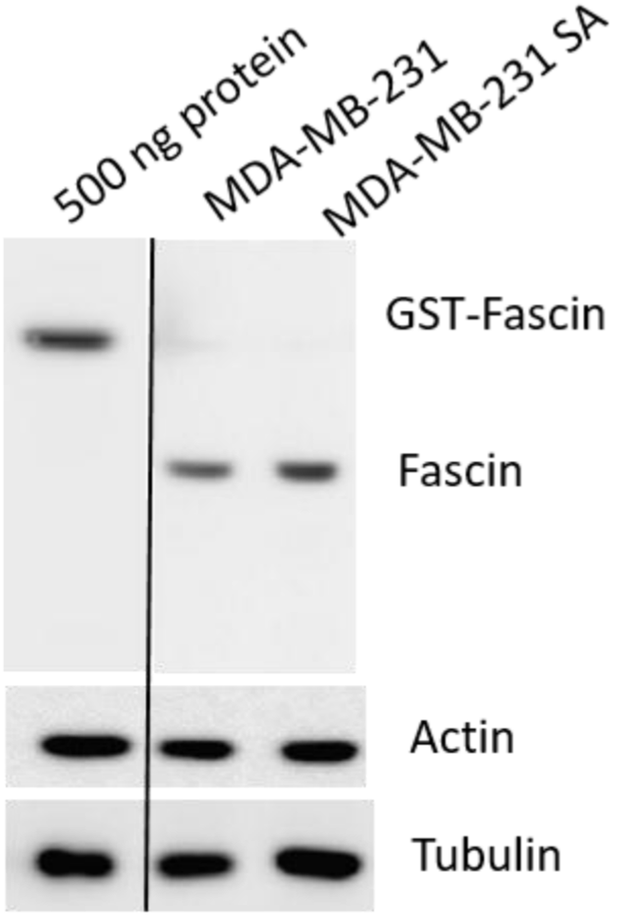

Supplementary Figure 2: Determination of fascin to actin and fascin to tubulin ratio in parental MDA-MB-231 and in in MDA-MB-231-SA cells. 500 ng protein standards of GST-fascin, actin and tubulin as well as 15 µg protein extract from parental MDA-MB-231 and in in MDA-MB-231-SA cells were analyzed by western-blotting employing antibodies against fascin, actin and tubulin.

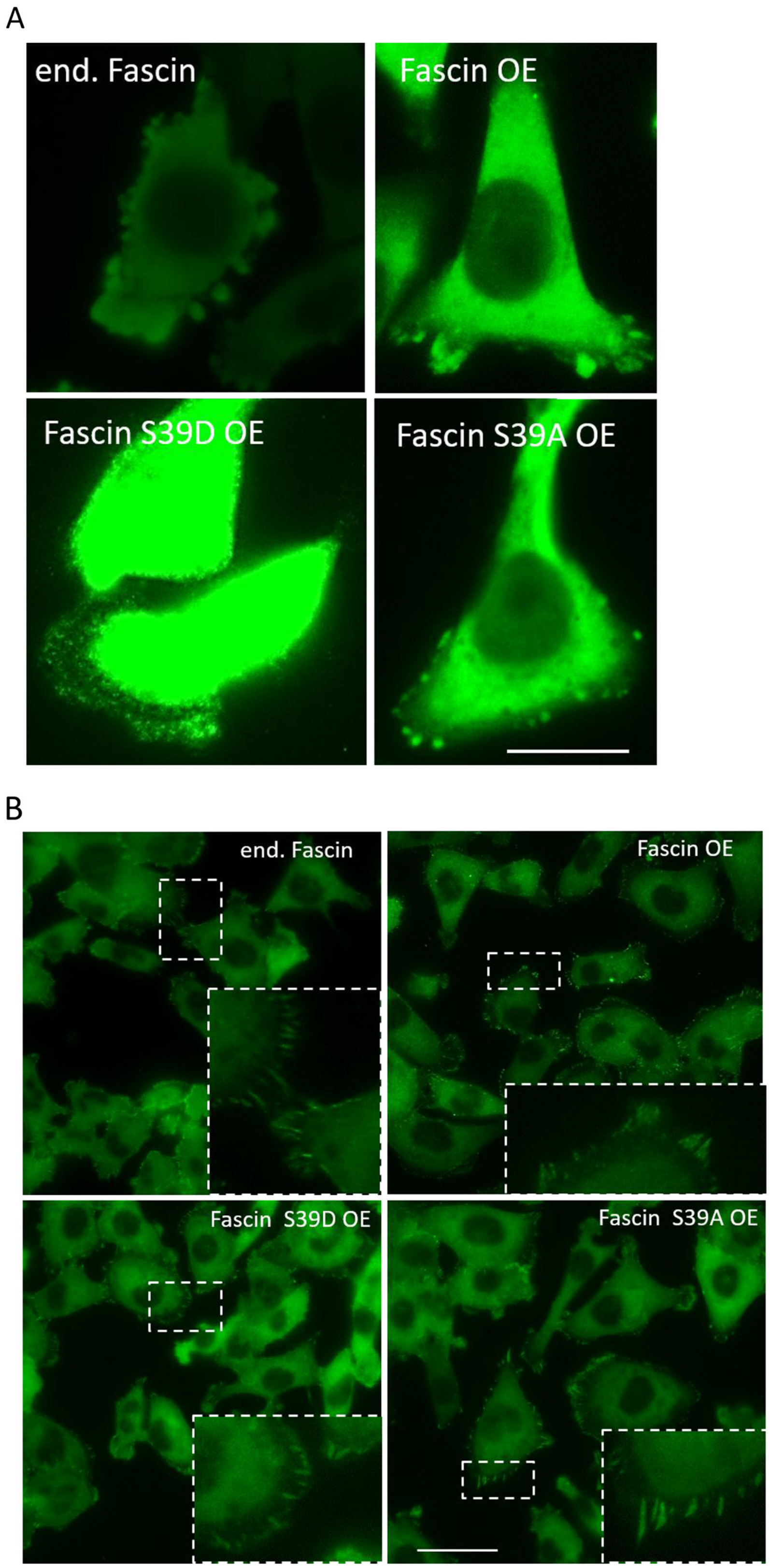

Supplementary Figure 3: Impact of fascin hyperexpression on elongation of filopodia. **(A)** To control that after hyperexpression fascin still is located in filopodia control MDA-MB-231 as well as fascin hyperexpressing cells were stained with an Alexa-fluor®488-coupled anti-fascin antibody. Bars: 10 µm. **(B)** To label filopodia, the cells were stained with an Alexa-fluor®488-coupled anti-VASP antibody.

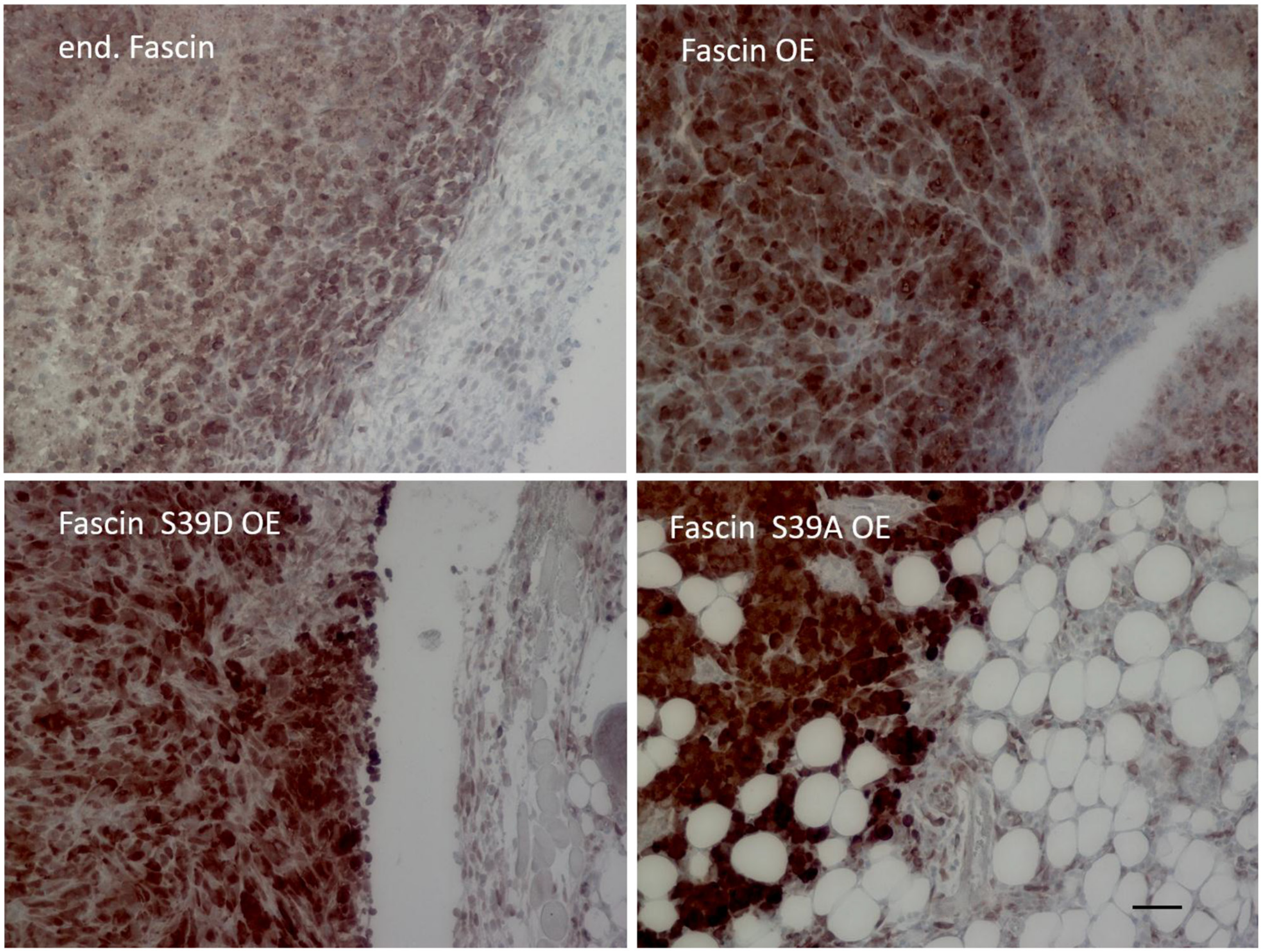

Supplementary Figure 4: Immunostain of fascin in primary tumors. Control and fascin hyperexpressing cells were injected subcutaneously between the scapulae of mice. The mice were sacrificed when the tumors had reached a size of 1.5 cm^2. The tumors were dissected, embedded in paraffin and stained with a fascin antibody. Brown colour indicate fascin expression. Fascin is mainly expressed in the tumors but also stroma cells show fascin expression. Bar: 50 µm.

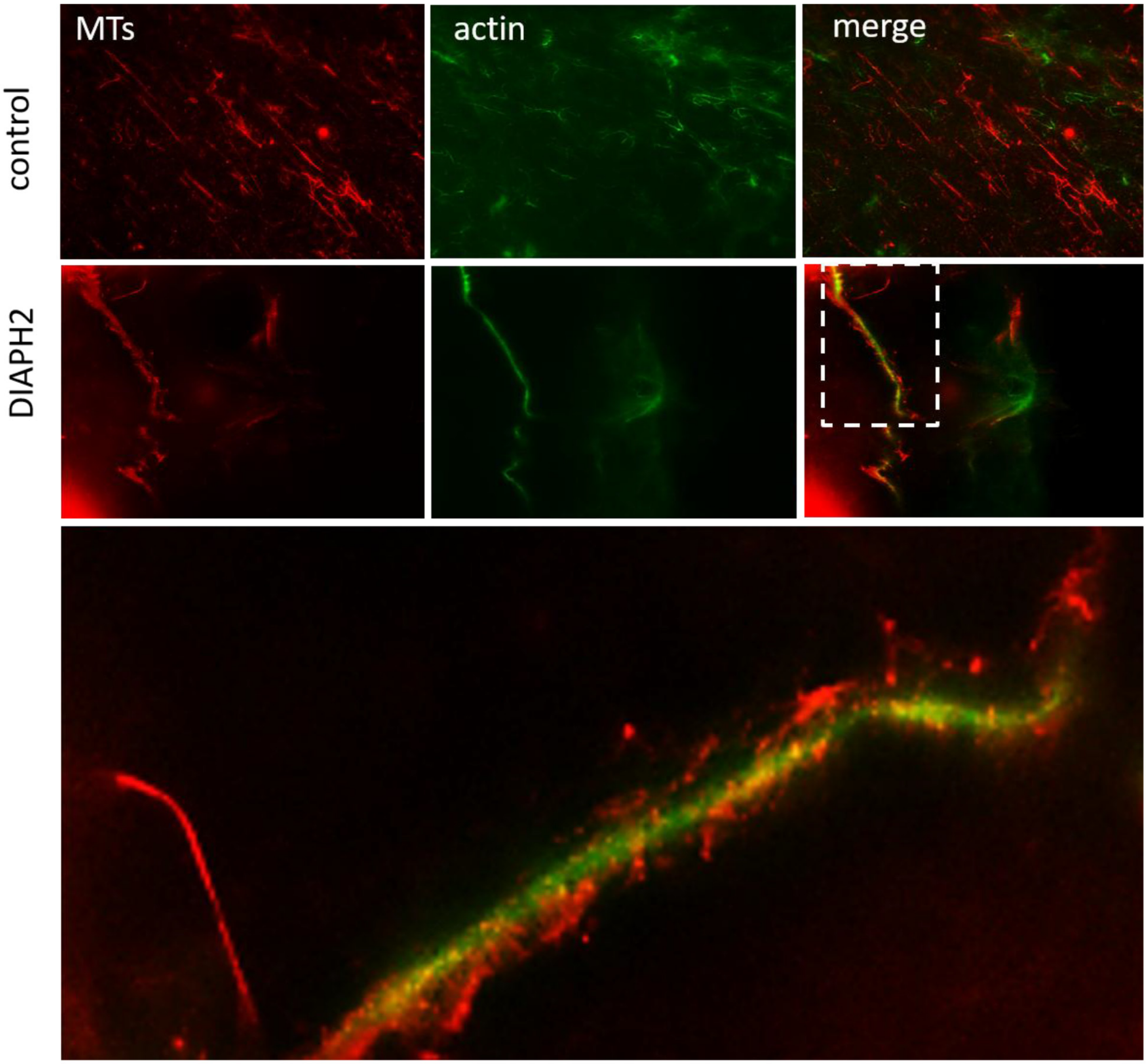

Supplementary Figure 5: DIAPH2 connects the actin with the tubulin cytoskeleton in a cell-free system. The sameprocedure as described in 7A was performed but instead of fascin purified 2 µg GST-DIAPH2 was employed.
